# Atrioventricular Nodal Blocking Agents and Adverse Outcomes Following Cardioversion at a Rural Hospital

**DOI:** 10.1002/joa3.70481

**Published:** 2026-10-23

**Authors:** Zach Caverley, Hsin‐Fang Li, Kateri J. Spinelli

**Affiliations:** ^1^ Providence Heart Clinic North Coast, Providence Oregon Cardiology Group Seaside Oregon USA; ^2^ Center for Cardiovascular Analytics, Research and Data Science (CARDS), Providence Heart Institute, Providence Research Network Portland Oregon USA

**Keywords:** atrioventricular blocking medication, bradyarrhythmia, cardioversion, conduction disorder, tachyarrhythmia

## Abstract

**Introduction:**

Direct current cardioversion (DCCV) is a well‐established procedure for terminating various tachyarrhythmias. Recommendations for pre‐operative adjustment of atrioventricular nodal blocking agents (AVNBA) to minimize bradyarrhythmia are lacking.

**Methods:**

We performed a retrospective analysis assessing safety and outcomes for our practice of holding AVNBA prior to DCCV. Adult patients with an elective DCCV from 10/01/2015 to 9/08/2023 were included and grouped into AVNBA held versus not held. Primary outcomes were perioperative bradyarrhythmia and 30 day bradyarrhythmia. Secondary outcomes included hypotension, 30 day tachyarrhythmia, ER visits, and other adverse events. Multilevel logistic regression models adjusted for demographics, comorbidities, and post‐discharge treatments were run.

**Results:**

The study included 247 procedures (68% male, average age 70.6 years), with 150 (61%) in the AVNBA not held group and 97 (39%) in the AVNBA held group. At 30 day follow up, the AVNBA not held group had higher rates of recurrent tachyarrhythmia (39% vs. 25%, *p* = 0.019). After risk adjustment, antiarrhythmic drug use predicted perioperative (aOR = 4.46; 95% CI = 1.68–11.89) and 30 day bradyarrhythmia (aOR = 2.81; 95% CI = 1.30–6.09). AVNBA holding was not predictive of either outcome. After risk adjustment, AVNBA holding was not a significant predictor of 30 day tachyarrhythmia recurrence; baseline antiarrhythmic drug use was associated with lower odds of recurrence (aOR = 0.38; 95% CI = 0.19–0.76).

**Conclusions:**

Holding AVNBA was not predictive of peri‐op or 30 day bradyarrhythmia after risk adjustment. Not holding AVNBA was associated with higher rates of recurrent tachyarrhythmia, but there was no association after risk adjustment. Our study reaffirms that elective DCCV is a safe and effective procedure, regardless of AVNBA holding status.

## Graphical abstract

This retrospective single‐center cohort study assessed safety and outcomes related to the practice of holding AVNBA prior to elective DCCV. Procedural‐level analyses comparing AVNBA holding 24 h prior to cardioversion showed that holding AVNBAs prior to elective DCCV was not associated with meaningful differences in short‐term arrhythmic safety outcomes.

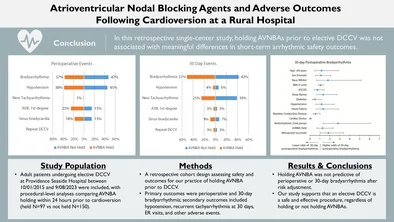

**Abbreviations**
AAD: antiarrhythmic drug
AF: atrial fibrillation
AFL: atrial flutter
ASCVD: atherosclerotic cardiovascular disease
AVN: atrioventricular node
AVNBA: atrioventricular nodal blocking agent
BB: beta blockers
DCCV: direct current cardioversion
ECG: electrocardiogram
EMR: electronic medical record
HR: heart rate
ICD: implantable Cardioverter‐Defibrillator
TEE: transesophageal echocardiogram

## 1 Introduction

The direct current cardioversion (DCCV) has been established as a safe and effective procedure for the restoration of normal rhythm in patients suffering from atrial fibrillation and atrial flutter [1]. Despite this, there remains a risk of postoperative conduction disturbances following shock delivery. A Danish registry analysis demonstrating 30 day bradyarrhythmia complications following the procedure ranged from 0.5% to 5.1%, with increased risk in older patients [2]. While this same study did not find antiarrhythmic drug (AAD) use increased the risk of bradyarrhythmia, use of rate lowering drugs like beta blockers (BB) did. Use of BB was high in this population (79%), underscoring the perceived need for careful rate control in these patients.

Consensus on preoperative medication management to optimize DCCV safety outcomes is lacking. A study out of Canada modified rate control agent use based on patient heart rate (HR) in the 48 h prior to DCCV to minimize the risk of postoperative bradyarrhythmia [3]. While this practice improved perioperative rate control and lowered the chance of post‐DCCV bradycardia (defined as HR < 50 bpm in that study), no significant bradyarrhythmia complications beyond low HR were reported. Additionally, although the authors described their protocol as “easily implementable,” the criteria for rate control medication dose adjustment carry the risk of patient and staff misinterpretation, which may limit implementation of the study's proposed framework in lower resource healthcare settings such as rural hospitals.

The current preoperative protocol for elective cardioversion at our rural hospital is simple: excluding AADs, patients are to hold all atrioventricular nodal blocking agents (AVNBA) 24 h prior to elective DCCV. This protocol was derived from a mechanistic understanding of drug therapy used for rate control and literature showing rare and significant postoperative conduction disturbances are predicted by a lower HR prior to DCCV [4]. While our protocol was reified by the aforementioned Danish registry findings [2], a large retrospective analysis on arrhythmic complications following DCCV did not find the use of BBs predicted bradyarrhythmia complications [5]. These conclusions have created the need for a more specific study to clarify the outcomes of our preoperative protocol.

To that end, we performed a retrospective analysis to evaluate if postoperative adverse arrhythmia outcomes varied between patients who held or continued AVNBA rate control medications in the 24 h prior to elective cardioversion. Our hypothesis was that the rate of adverse outcomes would not vary based on AVNBA use around the time of DCCV.

## 2 Methods

### 2.1 Patient Population and Data Source

This retrospective cohort study evaluated the association of AVNBA use on perioperative complications following DCCV. Patients aged 18 years and older who underwent elective DCCV from October 1, 2015, to September 8, 2023, at Providence Seaside Hospital were included. Elective DCCV was identified by CPT codes in the Epic electronic medical records (EMR). Patient data, including demographics, DCCV timing, diagnoses, adverse events, vital signs, electrocardiograms (ECGs), and provider notes, were obtained from the EMR via chart review. Comorbid clinical conditions were captured from the EMR using ICD‐10 and CPT codes (Supplemental Appendix), and the presence of PPM/ICD during DCCV was verified through chart review. Clinical information from Providence Astoria Heart Clinic and Ocean Beach Hospital was included to capture preoperative and follow‐up care.

### 2.2 Outcomes and Comparator Groups

The primary outcomes were perioperative bradyarrhythmia or conduction disturbance and postoperative bradyarrhythmia or conduction disturbance at 30 days. Secondary outcomes were other adverse events, including hypotension, 30 day tachyarrhythmia, and ER visits at various time points (perioperative, within 2 weeks of procedure, and within 30 days of procedure). Patients were identified by the AVNBA usage and type and placed into two groups for comparison: AVNBA Held versus AVNBA Not Held 24 h prior to elective DCCV procedure (hereby denoted as the “hold” and “no hold” groups, respectively). The clinical team implemented the practice change for holding AVNBAs in late 2020. To address potential selection bias, additional analyses were performed to examine demographic differences within the “not held” group before and after the practice change, and between “held” and “not held” group post‐2020.

### 2.3 Statistical Analysis

Patient demographics, cardiovascular comorbidities, procedure outcomes, adverse events, and follow‐up outcomes were summarized as descriptive statistics. Categorical data were presented as frequency (percentage). Continuous data were presented as mean (SD) or median (interquartile range [IQR]), as appropriate. Categorical variables were compared between groups using chi‐square test of association or Fisher's exact test, where appropriate. Continuous variables were compared between groups by two‐sample *t*‐test or Mann–Whitney Wilcoxon rank sum test, where appropriate. Because patients could have more than one procedure, data were analyzed at the procedure level rather than the patient level.

To evaluate the association between AVNBA usage in the 24 h prior to procedure and adverse events, multilevel logistic regression models were utilized to account for the within‐patient variability. These models were adjusted for demographics and cardiovascular comorbidities, with results presented as odds ratios (OR) and 95% confidence intervals (CI). Perioperative bradyarrhythmia, 30 day bradyarrhythmia, and 30 day tachyarrhythmia were treated as outcomes in the logistic regression, while adjusting for AVNBA holding, age, sex, body mass index (BMI), race, presence of a cardiac device, use of AADs in 2 week preop, baseline conduction disease, heart failure, hypertension, diabetes, sleep apnea, and atherosclerotic cardiovascular disease (ASCVD). Discharge metoprolol succinate use was additionally included as a covariate in the 30 day outcome models but not in the perioperative model, given its relevance to post‐discharge outcomes. Subgroup analyses were performed to evaluate the incidence of 30 day bradyarrhythmia in five subgroups: patients without a cardiac device, patients on AADs at baseline, patients with baseline conduction diseases, procedures with shock energy ≤ 200 J, and procedures with ≥ 2 shocks. All analyses were conducted using SAS Enterprise Guide 8.4. Significance was considered at *p* < 0.05.

## 3 Results

After exclusions, 247 procedures from 187 patients were included in the analysis with an average age of the patients being 70.6 years, and a majority of patients (68%) were male. The number of procedures per patient ranged from 1 to 5, with 77% undergoing one procedure, 15% undergoing two, 7% undergoing three, and 1% undergoing five procedures. The most common comorbidities were hypertension (74%), ASCVD (32%), and sleep apnea (32%) (Table 1). There were 150 procedures in the no hold group and 97 in the hold group. Comorbidities, clinical characteristics, and procedural characteristics were well balanced between groups. Exceptions included (but were not limited to) 22% higher metoprolol succinate use in the hold group (59% vs. 37%, *p* = 0.001) and 8% higher flecainide use in the hold group (14% vs. 6%, *p* = 0.042), and slightly higher preoperative blood pressure (BP) in the hold group (systolic BP: 127.3 vs. 122.3; diastolic BP: 77.8 vs. 73.7) (Table 2). Transesophageal echocardiogram (TEE) usage during the procedure was 13% higher in the no hold group (21% vs. 8%, *p* = 0.012) (Table 3). Although TEE usage differed, the average procedure time and anesthetic dose used between groups were similar (Table 3). Daily total doses of metoprolol in the time points leading up to and after the procedure are reported in Table S1. Because the practice changes of holding AVNBAs occurred in late 2020, the no hold group was concentrated earlier in the study time frame, and the hold group was concentrated later in the study period (Table S2). Prior to this practice change, there were three cases where AVNBA were held as an independent choice made by the clinician due to a perceived higher risk of perioperative events in those specific cases. There were minimal differences in patient demographics in the no hold group before and after 2020 (Table S3), and no differences between the hold and no hold groups after 2020 (Table S4).

**TABLE 1:** Patient characteristics.

Variable	All procedures (*N* = 247)	AVNBA not held (*N* = 150)	AVNBA held (*N* = 97)	*p*
Age, years	70.6 (9.0)	69.8 (9.6)	71.7 (8.0)	0.1210
Sex, female	78 (32%)	49 (33%)	29 (30%)	0.6474
BMI	33.4 (7.5)	33.7 (7.7)	32.9 (7.2)	0.4461
Race				0.2322
American Indian/Alaska Native	3 (1%)	1 (1%)	2 (2%)	
Asian	1 (0.4%)	1 (1%)	0 (0%)	
Other	4 (2%)	4 (3%)	0 (0%)	
White	224 (97%)	132 (96%)	92 (98%)	
Ethnicity, % Hispanic	18 (7%)	13 (9%)	5 (5%)	0.2997
Insurance type				0.394
Commercial	37 (15%)	19 (13%)	18 (19%)	
Medicaid/low‐income	6 (2%)	5 (3%)	1 (1%)	
Medicare	193 (78%)	118 (79%)	75 (77%)	
Self‐pay	2 (1%)	2 (1%)	0 (0%)	
Other	9 (4%)	6 (4%)	3 (3%)	
Baseline Comorbidities				
Heart failure	59 (24%)	39 (26%)	20 (21%)	0.3327
Hypertension	184 (74%)	113 (75%)	71 (73%)	0.7067
Diabetes mellitus	38 (15%)	26 (17%)	12 (12%)	0.2912
Sleep apnea	79 (32%)	49 (33%)	30 (31%)	0.7748
Prior or multiple cardioversion	18 (7%)	12 (8%)	6 (6%)	0.5921
PPM/ICD present	33 (13%)	20 (13%)	13 (13%)	1
ASCVD	79 (32%)	43 (29%)	36 (37%)	0.1645

*Note:* Data presented as mean (sd) or *n* (%) based on non‐missing values.

Abbreviations: ASCVD = atherosclerotic cardiovascular disease, AVNBA = atrioventricular nodal blocking agents, BMI = body mass index, ICD = implantable cardioverter‐defibrillator, PPM = permanent pacemaker.

**TABLE 2:** Preoperative clinical characteristics.

Variable	All procedures (*N* = 247)	AVNBA not held (*N* = 150)	AVNBA held (*N* = 97)	*p*
Afib, < 1 month prior to procedure	45 (18%)	32 (22%)	13 (13%)	0.1148
Afib, > 1 month prior to procedure	200 (82%)	116 (78%)	84 (87%)	0.0541
**30 days Preoperative**				
Heart rate	87.3 (19.5)	89.1 (19.5)	84.7 (19.1)	0.0820
Blood pressure (systolic)	124.4 (17.1)	122.3 (17.6)	127.3 (16)	0.0278
Blood pressure (diastolic)	75.3 (12.4)	73.7 (13.1)	77.8 (11.1)	0.0109
Electrocardiogram				
Atrial fibrillation	178 (72%)	114 (76%)	64 (66%)	0.0865
Atrial flutter	69 (28%)	36 (24%)	33 (34%)	0.0865
Atrial tachycardia	3 (1%)	1 (1%)	2 (2%)	0.5631
LBBB	7 (3%)	5 (3%)	2 (2%)	0.7075
RBBB	24 (10%)	15 (10%)	9 (9%)	0.8517
IVCD	47 (19%)	27 (18%)	20 (21%)	0.6086
Prolonged QTc	23 (9%)	17 (11%)	6 (6%)	0.1740
LAFB	22 (9%)	13 (9%)	9 (9%)	0.8691
LPFB	2 (1%)	1 (1%)	1 (1%)	1
Ventricular rate				0.8023
Bradycardia (40–59 BPM)	10 (4%)	6 (4%)	4 (4%)	
Normal (60–99 BPM)	178 (72%)	106 (70%)	72 (74%)	
Tachycardia (≥ 100 BPM)	59 (24%)	38 (25%)	21 (22%)	
**Immediate Preoperative**				
Heart rate	84.1 (17.5)	83 (16)	85.8 (19.5)	0.2224
Blood pressure (systolic)	131.4 (16.2)	129.7 (16.3)	133.9 (15.9)	0.0463
Blood pressure (diastolic)	79.4 (13)	78.0 (13.6)	81.5 (11.9)	0.0360
Electrocardiogram readings				
Atrial fibrillation	177 (72%)	111 (74%)	66 (68%)	0.3102
Atrial flutter	75 (30%)	41 (27%)	34 (35%)	0.1977
Junctional arrhythmia	2 (1%)	1 (1%)	1 (1%)	1
LBBB	8 (3%)	5 (3%)	3 (3%)	1
RBBB	28 (11%)	15 (10%)	13 (13%)	0.4102
IVCD	48 (19%)	26 (17%)	22 (23%)	0.2997
Prolonged QTc	30 (12%)	19 (13%)	11 (11%)	0.7553
LAFB	24 (10%)	15 (10%)	9 (9%)	0.8517
LPFB	3 (1%)	0 (%)	3 (3%)	0.0594
Ventricular rate				0.0311
Bradycardia (40–59 BPM)	10 (4%)	10 (7%)	0 (0%)	
Normal (60–99 BPM)	181 (75%)	107 (73%)	74 (77%)	
Tachycardia (≥ 100 BPM)	51 (21%)	29 (20%)	22 (23%)	
Baseline conduction disease	113 (46%)	65 (43%)	48 (49%)	0.3433
**Medications, 2 weeks Pre‐procedure**				
AVNB Agents				
Metoprolol tartrate	49 (20%)	30 (20%)	19 (20%)	1
Metoprolol succinate	113 (46%)	56 (37%)	57 (59%)	0.0011
Bisoprolol	1 (0.4%)	0 (0%)	1 (1%)	0.3927
Carvedilol	28 (11%)	14 (9%)	14 (14%)	0.2248
Diltiazem	23 (9%)	18 (12%)	5 (5%)	0.0770
Digoxin	7 (3%)	3 (2%)	4 (4%)	0.4379
Antiarrhythmics				
Amiodarone	166 (67%)	102 (68%)	64 (66%)	0.7821
Sotalol	4 (2%)	4 (3%)	0 (0%)	0.1568
Tikosyn	2 (1%)	2 (1%)	0 (0%)	0.5211
Propafenone	4 (2%)	1 (1%)	3 (3%)	0.3029
Flecainide	23 (9%)	9 (6%)	14 (14%)	0.0415
Dronedarone	2 (1%)	2 (1%)	0 (0%)	0.5211

*Note:* Data presented as mean (sd) or *n* (%) based on non‐missing values.

Abbreviations: Afib = atrial fibrillation, AVNB = atrioventricular nodal blocking, AVNBA = atrioventricular nodal blocking agents, BPM = beats per minute, IVCD = intraventricular conduction delay, LAFB = left anterior fascicular block, LBBB = left bundle branch block, LPFB = left posterior fascicular block, QTc = QT Interval (corrected), RBBB = right bundle branch block.

**TABLE 3:** Procedural and perioperative outcomes.

Variable	All procedures (*N* = 247)	AVNBA not held (*N* = 150)	AVNBA held (*N* = 97)	*p*
**Procedural Outcomes**				
Procedure success	237 (96%)	145 (97%)	92 (95%)	0.5203
Paddle/electrode position*				0.5631
Anterior/lateral	3 (1%)	1 (1%)	2 (2%)	
Anterior/posterior	244 (99%)	149 (99%)	95 (98%)	
Number of shocks				0.5721
1	220 (90%)	131 (89%)	89 (92%)	
2	15 (6%)	11 (7%)	4 (4%)	
3	10 (4%)	6 (4%)	4 (4%)	
Joules used for shock (J)	213.1 (51.4)	225.8 (59.8)	193.5 (24.3)	< 0.0001
Propofol quantity (mg)	70 (60, 100)	80 (60, 120)	70 (60, 95)	0.0518
Procedure time, minutes	121 (110, 137)	120 (110, 142)	122 (109, 133)	0.7088
TEE usage	39 (16%)	31 (21%)	8 (8%)	0.0116
Concomitant procedures				
Temporary TC pacing	1 (0.4%)	0 (0%)	1 (1%)	0.3927
Initiation of ACLS	1 (0.4%)	0 (0%)	1 (1%)	0.3927
Other^^^	2 (1%)	0 (0%)	2 (2%)	0.1533
Inpatient hospitalization	2 (1%)	2 (1%)	0 (0%)	0.5211
LOS, hours	56.5	56.5	NA	
**Postoperative Vitals**				
Heart rate				
Highest HR	67.8 (14.5)	66.9 (14.7)	69.3 (14.2)	0.2245
Lowest HR	59.6 (10.9)	58.2 (10.4)	61.7 (11.3)	0.0148
Blood pressure				
Highest systolic BP	125.3 (16.3)	124.6 (16.6)	126.3 (16)	0.4493
Lowest systolic BP	104.7 (15.4)	103.7 (15.7)	106.3 (14.8)	0.2085
Highest diastolic BP	76.7 (12.6)	76 (12)	77.9 (13.4)	0.2456
Lowest diastolic BP	59.6 (11.4)	58.4 (11.2)	61.4 (11.6)	0.0498
Electrocardiogram readings				
Normal sinus rhythm	120 (48%)	69 (46%)	51 (53%)	0.3618
Sinus bradycardia	95 (38%)	60 (40%)	35 (36%)	0.5930
Sinus tachycardia	2 (1%)	1 (1%)	1 (1%)	1
1st degree AVB	102 (41%)	57 (38%)	45 (46%)	0.2337
Atrial fibrillation	5 (2%)	4 (3%)	1 (1%)	0.6510
Junctional arrhythmia	4 (2%)	0 (0%)	4 (4%)	0.0229
Other SVT unspecified	2 (1%)	2 (1%)	0 (0%)	0.5211
LBBB	6 (2%)	4 (3%)	2 (2%)	1
RBBB	24 (10%)	12 (8%)	12 (12%)	0.2773
IVCD	31 (13%)	19 (13%)	12 (12%)	1
Prolonged QTc	37 (15%)	25 (17%)	12 (12%)	0.4656
LAFB	25 (10%)	13 (9%)	12 (12%)	0.3906
LPFB	1 (0.4%)	0 (0%)	1 (1%)	0.3927
Ventricular Rate (highest)				0.8290
Severe bradycardia (< 40 BPM)	1 (0.4%)	1 (1%)	0 (0%)	
Bradycardia (40–59 BPM)	88 (36%)	52 (35%)	36 (37%)	
Normal (60–99 BPM)	156 (63%)	96 (64%)	60 (62%)	
Tachycardia (≥ 100 BPM)	2 (1%)	1 (1%)	1 (1%)	
Ventricular Rate (lowest)				0.2948
Severe bradycardia (< 40 BPM)	2 (1%)	2 (1%)	0 (0%)	
Bradycardia (40–59 BPM)	104 (43%)	67 (46%)	37 (39%)	
Normal (60–99 BPM)	135 (56%)	79 (53%)	56 (60%)	
Tachycardia (≥ 100 BPM)	1 (0.4%)	0 (0%)	1 (1%)	
**Discharge Medications**				
AVNB agents				
Metoprolol tartrate	44 (18%)	27 (18%)	17 (18%)	1
Metoprolol succinate	111 (45%)	57 (38%)	54 (56%)	0.0087
Bisoprolol	1 (0.4%)	0 (0%)	1 (1%)	0.3927
Carvedilol	27 (11%)	15 (10%)	12 (12%)	0.6768
Diltiazem	14 (6%)	10 (7%)	4 (4%)	0.5749
Digoxin	3 (1%)	1 (1%)	2 (2%)	0.5631
Antiarrhythmics				
Amiodarone	172 (70%)	104 (70%)	68 (70%)	1
Sotalol	5 (2%)	5 (3%)	0 (0%)	0.1598
Tikosyn	4 (2%)	4 (3%)	0 (0%)	0.1568
Propafenone	9 (4%)	2 (1%)	7 (7%)	0.0307
Flecainide	27 (11%)	13 (9%)	14 (14%)	0.2096
Dronedarone	6 (2%)	4 (3%)	2 (2%)	1
No AVNB agents or Antiarrhythmic	3 (1%)	3 (2%)	0 (0%)	0.2816

*Note:* Data presented as mean (sd), median [interquartile range] or *n* (%) based on non‐missing values.

Abbreviations: ACLS = advanced cardiac life support, AVB = atrioventricular block, AVNBA = atrioventricular nodal blocking agents, BP = blood pressure, BPM = beats per minute, HR = heart rate, IVCD = intraventricular conduction delay, LAFB = left anterior fascicular block, LBBB = left bundle branch block, LOS = length of stay, LPFB = left posterior fascicular block, QTc = QT Interval (corrected), RBBB = right bundle branch block, SVT = supraventricular tachycardia, TC = transcutaneous, TEE = transesophageal echocardiogram.

* Patients may be counted more than once if they had multiple shocks.

^^^ Other action taken was the administration of IV fluids for hypotension.

For the perioperative, 2‐week, and 30 day follow up assessments, several findings differed between groups. In the perioperative setting, the hold group tended to have a higher rate of new 1st degree AV block (25% vs. 15%, *p* = 0.06), although without statistical significance. There were no episodes of advanced atrioventricular block (AVB) after cardioversion in either group (Table 4). The rate of perioperative hypotension was 7% higher in the no hold group (45% vs. 38%), although this difference did not achieve statistical significance. The incidence of recurrent tachyarrhythmia—atrial fibrillation (AF) and atrial flutter (AFL) – was 10% greater in the no hold group (33% vs. 23%, *p* = 0.072) within 2 weeks following cardioversion and increased to 14% higher (39% vs. 25%, *p* = 0.019) at the 30 day follow up. The presence of any given bradyarrhythmia at 30 day follow‐up was proportionally higher in the hold group (51% vs. 42%, *p* = 0.189). Any cause ER visit within 30 days of the procedure was 6% higher in the no hold group (9% vs. 3%, *p* = 0.072).

**TABLE 4:** Adverse events.

Variable	All procedures (*N* = 247)	AVNBA not held (*N* = 150)	AVNBA held (*N* = 97)	*p*
**Postoperative**				
Bradyarrhythmia	107 (43%)	71 (47%)	36 (37%)	0.1134
New conduction disease				
Sinus‐bradycardia	39 (16%)	22 (15%)	17 (18%)	0.5473
Junctional arrhythmia	2 (1%)	0 (0%)	2 (2%)	0.1533
AVB, 1st degree	47 (19%)	23 (15%)	24 (25%)	0.0658
RBBB	1 (0.4%)	1 (1%)	0 (0%)	1
IVCD	3 (1%)	1 (1%)	2 (2%)	0.5631
Prolonged QTc	11 (4%)	9 (6%)	2 (2%)	0.2091
LAFB	3 (1%)	2 (1%)	1 (1%)	1
Hypotension	104 (42%)	67 (45%)	37 (38%)	0.3106
New Tachyarrhythmia	1 (0.4%)	0 (0%)	1 (1%)	0.2103
Sinus‐tachycardia	1 (0.4%)	0 (0%)	1 (1%)	0.2103
Oxygen desaturation	7 (3%)	5 (3%)	2 (2%)	0.3838
Rebound hypertension	23 (9%)	10 (7%)	13 (13%)	0.1147
**At 2 Weeks Post‐procedure**				
Repeat elective DCCV	3 (1%)	3 (2%)	0 (0%)	0.2816
Bradyarrhythmia	91 (37%)	50 (33%)	41 (42%)	0.1551
New conduction disease				
Sinus‐bradycardia	13 (5%)	7 (5%)	6 (6%)	0.7716
Junctional arrhythmia	2 (1%)	2 (1%)	0 (0%)	0.5211
AVB, 1st degree	5 (2%)	2 (1%)	3 (3%)	0.3839
RBBB	1 (0.4%)	1 (1%)	0 (0%)	1
IVCD	2 (1%)	2 (1%)	0 (0%)	0.5211
Prolonged QTc	4 (2%)	2 (1%)	2 (2%)	0.6467
LAFB	3 (1%)	2 (1%)	1 (1%)	1
LPFB	2 (1%)	1 (1%)	1 (1%)	1
Hypotension	7 (3%)	5 (3%)	2 (2%)	1
New Tachyarrhythmia	72 (29%)	50 (33%)	22 (23%)	0.0720
Atrial fibrillation	54 (22%)	41 (27%)	13 (13%)	0.0114
Atrial flutter	20 (8%)	11 (7%)	9 (9%)	0.6365
Rebound hypertension	20 (8%)	15 (10%)	5 (5%)	0.2335
**At 30 days Post‐procedure**				
Repeat elective DCCV	8 (3%)	5 (3%)	3 (3%)	1
Bradyarrhythmia	112 (45%)	63 (42%)	49 (51%)	0.1892
New conduction disease				
Sinus‐bradycardia	20 (8%)	11 (7%)	9 (9%)	0.6365
Junctional arrhythmia	2 (1%)	2 (1%)	0 (0%)	0.5211
AV Block, 1st degree	7 (3%)	4 (3%)	3 (3%)	1
LBBB	1 (0.4%)	0 (0%)	1 (1%)	0.3927
RBBB	1 (0.4%)	1 (1%)	0 (0%)	1
IVCD	3 (1%)	3 (2%)	0 (0%)	0.2816
Prolonged QTc	4 (2%)	2 (1%)	2 (2%)	0.6284
LAFB	4 (2%)	2 (1%)	2 (2%)	0.6284
LPFB	2 (1%)	1 (1%)	1 (1%)	1
Hypotension	13 (5%)	9 (6%)	4 (4%)	0.5757
New Tachyarrhythmia	83 (34%)	59 (39%)	24 (25%)	0.0194
Atrial fibrillation	63 (26%)	49 (33%)	14 (14%)	0.0016
Atrial flutter	25 (10%)	14 (9%)	11 (11%)	0.6680
Rebound hypertension	49 (20%)	29 (19%)	20 (21%)	0.8706
ER visit (within 30d post procedure)	17 (7%)	14 (9%)	3 (3%)	0.0724

*Note:* Data presented as *n* (%) based on non‐missing values.

Abbreviations: AVB = atrioventricular block, AVNB = atrioventricular nodal blocking, AVNBA = atrioventricular nodal blocking agents, DCCV = direct current cardioversion, ER = emergency room, IVCD = intraventricular conduction delay, LAFB = left anterior fascicular block, LBBB = left bundle branch block, LOS = length of stay, LPFB = left posterior fascicular block, QTc = QT Interval (corrected), RBBB = right bundle branch block.

Several subgroup analyses were performed, including procedures of patients without a cardiac device meaning a pacemaker or implantable cardioverter‐defibrillator (ICD), procedures of patients taking an AAD, and procedures of patients with baseline cardiac conduction disease. When restricting the analysis to procedures without a cardiac device, the incidence of bradyarrhythmia was 10% higher in the hold group (58% vs. 48%, *p* = 0.128) (Table 5). There were similar findings when looking at patients taking any AAD, with an 11% higher occurrence in the hold group (56% vs. 45%, *p* = 0.153). For cardioversion procedures that had more or less than 200 J for the shock energy, there was a 12% higher proportion of bradyarrhythmia within 30 days if ≤ 200 J was used (47% vs. 35%, *p* = 0.177). Receiving more or less than 2 shocks did not appear to affect the incidence of bradyarrhythmia.

**TABLE 5:** Subgroup analyses of bradyarrhythmia at 30 days postoperative.

Variable	AVNBA not held	AVNBA held	*p*
**Patients without PPM/ICD during DCCV**			
Number of procedures	130	84	
Number (%) of procedures with bradyarrhythmia at 30 days	62 (48%)	49 (58%)	0.1282
**Patients with any antiarrhythmics 2 weeks preop**			
Number of procedures	120	81	
Number (%) of procedures with bradyarrhythmia at 30 days	54 (45%)	45 (56%)	0.1528
**Patients with baseline conduction diseases**			
Number of procedures	65	48	
Number (%) of procedures with bradyarrhythmia at 30 days	26 (40%)	22 (46%)	0.5680
**Shock Energy**			
	**Joules ≤ 200**	**Joules > 200**	
Number of procedures	203	43	
Number (%) of procedures with bradyarrhythmia at 30 days	96 (47%)	15 (35%)	0.1770
	**Shocks < 2**	**Shocks ≥ 2**	
Number of procedures	220	25	
Number (%) of procedures with bradyarrhythmia at 30 days	99 (45%)	12 (48%)	0.8338

*Note:* Data presented as *n* (%) based on non‐missing values.

Abbreviations: AVNBA = atrioventricular nodal blocking agents, DCCV = direct current cardioversion, ICD = implantable cardioverter‐defibrillator, PPM = permanent pacemaker.

In the regression analysis for the perioperative period, AAD use at baseline best predicted bradyarrhythmia (aOR = 4.46; 95% CI = 1.68, 11.89), followed by age > 60 years (aOR = 3.39; 95% CI = 1.08, 10.69), and heart failure at baseline (aOR = 2.60; 95% CI = 1.03, 6.57) (Figure 1). Having baseline conduction disease did not appear to be predictive for perioperative bradyarrhythmia. At 30 days, AAD use again best predicted bradyarrhythmia (aOR = 2.81; 95% CI = 1.30, 6.09) (Figure 1). Discharge metoprolol succinate use was also associated with higher odds of 30 day bradyarrhythmia compared with non‐use (aOR = 1.85; 95% CI = 1.02, 3.37), while AVNBA holding (aOR = 1.27; 95% CI = 0.7, 2.32) itself was not predictive of this outcome. In the adjusted model evaluating 30 day tachyarrhythmia recurrence, AVNBA holding was not a significant predictor (aOR = 0.54; 95% CI = 0.29, 1.01), while baseline AAD use was associated with lower odds of recurrence (aOR = 0.38; 95% CI = 0.19, 0.76) (Figure 2). Unsurprisingly, having a cardiac device in place significantly reduced the likelihood of bradyarrhythmia (aOR = 0.04 perioperatively and aOR = 0.02 at 30 days) (Figure 1).

**FIGURE 1:**
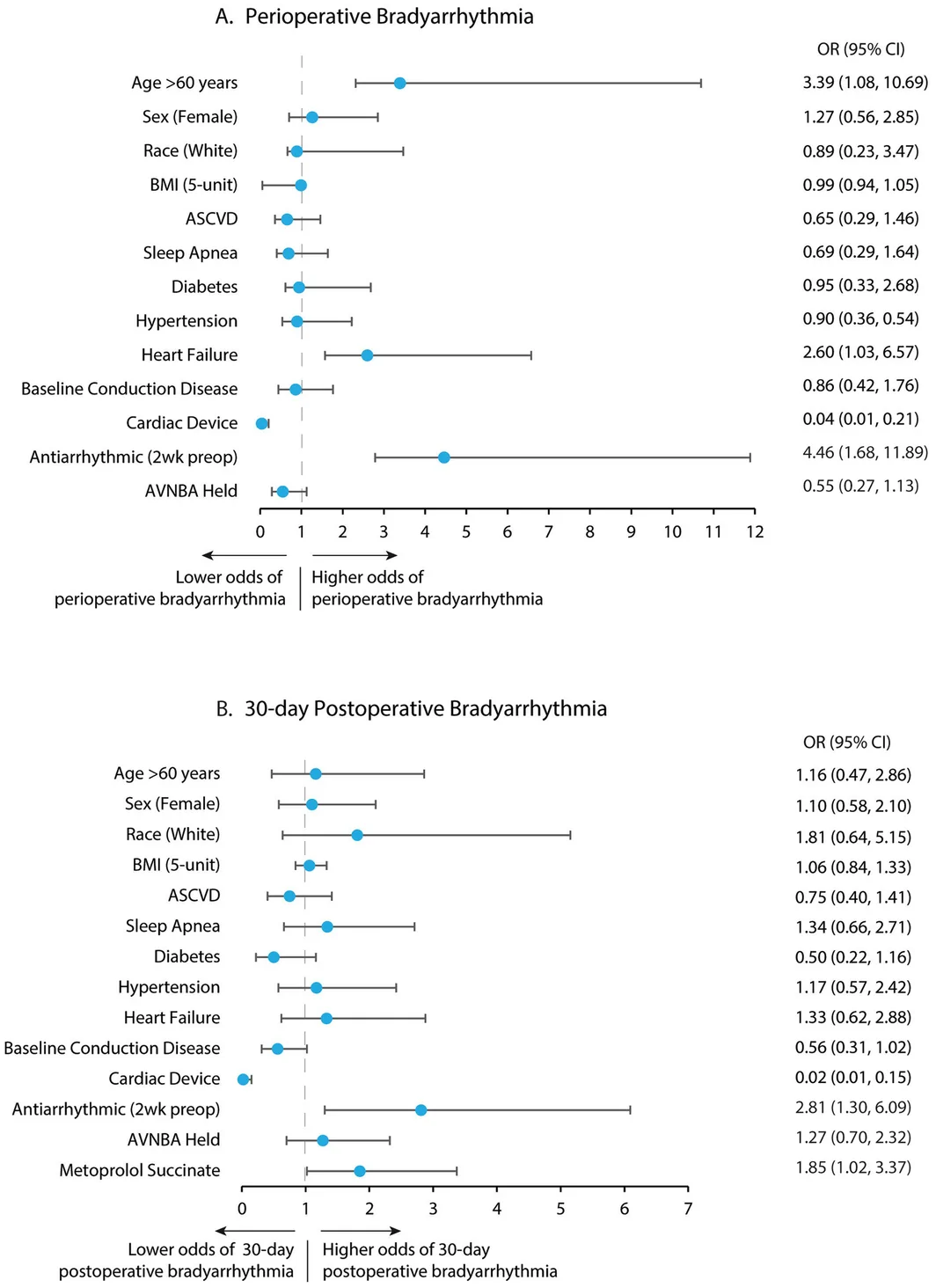
Predictors of (A) perioperative bradyarrhythmia and (B) 30 day postoperative bradyarrhythmia. Odds ratios and 95% confidence intervals are shown. ASCVD = atherosclerotic cardiovascular disease, AVNBA = atrioventricular nodal blocking agents, BMI = body mass index.

**FIGURE 2:**
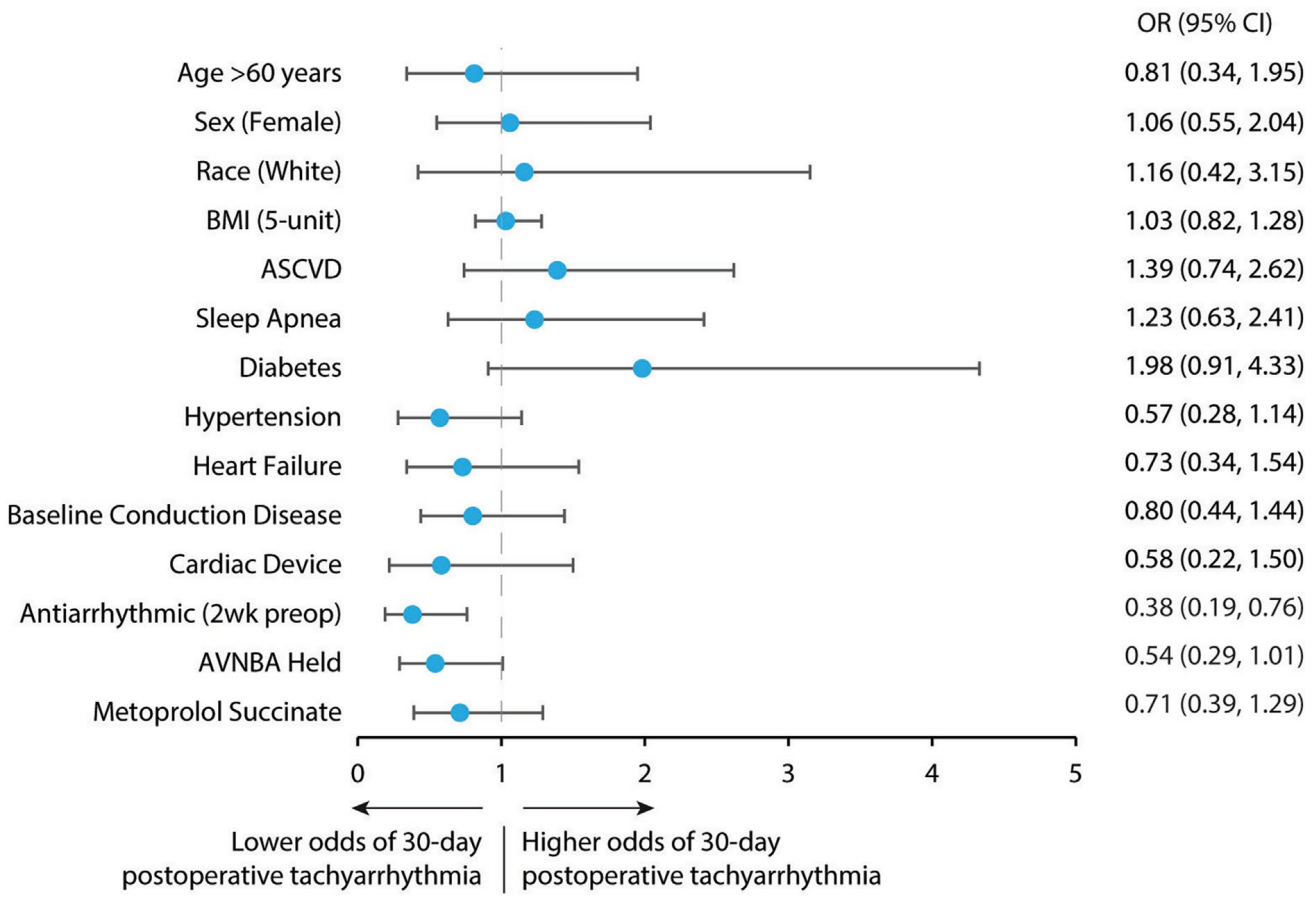
Predictors of 30 day postoperative tachyarrhythmia. Odds ratios and 95% confidence intervals are shown. ASCVD = atherosclerotic cardiovascular disease, AVNBA = atrioventricular nodal blocking agents, BMI = body mass index.

## 4 Discussion

Our results demonstrate that in the perioperative and 30 days follow up periods, the incidence of bradyarrhythmia and cardiac conduction disease did not significantly differ if AVNBA were held 24 h beforehand. Perioperative cardiac conduction disease was proportionally higher in the hold group, but these were often relatively benign findings such as 1st degree AV block. In fact, only three patients required actions like temporary pacing or IV fluid administration perioperatively which would limit attempts to narrow this analysis to events requiring an intervention. The incidence of tachyarrhythmia such as AF was significantly higher at 30 day follow up in the no hold group. Subgroup analysis showed a higher proportion of bradyarrhythmia in the hold group among procedures for patients without a cardiac device and patients taking any AAD. Regression analyses revealed AAD use was a significant predictor of bradyarrhythmia perioperatively and at 30 days, whereas advanced age and heart failure were only predictive of bradyarrhythmia in the perioperative period, and AVNBA holding was not predictive at either time point.

Our analysis is consistent with other studies showing increased risk of conduction disturbances following DCCV in older patients [2], but differs regarding AAD predicting bradyarrhythmia while AVNBAs used for rate control (like beta blockers) were not found to be a significant predictor. As mentioned in the introduction, another retrospective study also concluded BB use was not predictive of bradyarrhythmia complications [5]. It is possible the transient withholding of these agents (only 24 h prior to procedure) explains the lack of significant difference. A similar percentage of patients were discharged after DCCV on AVNBAs compared to the percentage taking AVNBAs 2 weeks prior in both groups, which indicates the postoperative changes were so trivial as to not necessitate large changes for prescribed medications.

Antiarrhythmic drug (AAD) use predicting bradyarrhythmia does not come as a surprise in this study given interruption of conduction through the sinoatrial and AV nodes is a known mechanistic property of many AADs [6]. The use of these drugs was quite common for patients in this study—with a notable preference for amiodarone which was in use during 67% of procedures. Although the proportion of Class IC agent use was significantly different between groups, the raw numbers for both Flecainide and Propafenone were quite low and, therefore, unlikely to have a significant effect on the odds ratio for bradyarrhythmia found in the regression analyses.

The 2‐week and 30 day incidence of bradyarrhythmia being proportionally higher in the hold group contrasted with the higher rate of recurrence of tachyarrhythmia in those that did not hold AVNBAs raises suspicion that the outcomes may be influenced by clinician choice and selection bias. Patients with a known history of bradyarrhythmia and conduction disease would be more prone to have lower doses of AVNBAs prescribed, while patients with particularly fast and burdensome tachyarrhythmias would be prescribed more AVNBAs at higher doses. For medications like metoprolol (both tartrate and succinate), the average dose was often the same or higher before and after DCCV in the no hold group compared to the hold group, which would seem to support such a hypothesis (Table S1). Another possible explanation is the higher rate of metoprolol succinate at discharge for the hold group, which was an independent predictor for 30 day postoperative bradyarrhythmia. It is ultimately unclear how much clinician choice regarding rate control prescriptions influenced the results, particularly given other rate control medications were either numerically low or not significantly different between groups outside of metoprolol succinate postoperatively.

The rate of ER visits in the no hold group was 6% higher than in the hold group. A qualitative review revealed roughly 13 out of 27 ER visits for the no hold group were due to cardiac diagnoses, which included AF—consistent with the higher rate of tachyarrhythmia recurrence in the no hold group already mentioned. It is ultimately indeterminate what the clinical significance of this finding is, given there were no ER visits or hospitalizations found to be specifically due to hypotension or bradyarrhythmia, as one might expect when continuing AVNBAs uninterrupted. There was one case that had a diagnosis of tremors and low HR, but this was determined to be due to amiodarone use, which was discontinued at that time.

Unveiling a larger difference in bradyarrhythmia when restricting the analysis to patients without cardiac devices is unsurprising, as bradyarrhythmia is the primary indication for pacemaker implantation [7]. This subgroup was fairly large—only excluding 33 procedures—and therefore lowers concerns that a small subgroup would superficially inflate statistical significance.

Our study has several limitations. Because the clinical practice change of holding or continuing AVNBA before cardioversion for atrial arrhythmias in our institution occurred in late 2020 without a specific date of implementation, the hold and no‐hold groups were largely drawn from different time periods, as shown in Table S2. This may influence the baseline characteristics and outcomes of this study and should be considered when interpreting the results and conclusions. Furthermore, the retrospective study design means causality cannot be attributed to holding or continuing AVNBAs for any given outcome. All outcomes data were collected via chart review, and while we followed a standardized method and definitions for both reviewers for data collection, we cannot rule out some degree of missingness if clinic staff failed to chart certain information or did not follow protocol. Additionally, subgroup analyses often face challenges, such as smaller sample sizes leading to diminished statistical power and unreliable results [8]. In our study, subgroups like procedures of patients with baseline conduction diseases may yield false positives and negatives due to the reduced sample size and higher variability. Selection bias may arise if subgroups are defined post hoc on variables that are unevenly distributed, which might skew the associations observed. Since subgroup analyses are typically exploratory and not pre‐specified, findings should be considered hypothesis‐generating rather than conclusive [9, 10]. As already stated, despite the healthcare center used in this analysis having a protocol in place for holding AVNBAs prior to DCCV, we cannot rule out some degree of clinician decision‐making confounding the results—a concern best exemplified by several patients not having documented instructions to hold AVNBAs after policy implementation. It is entirely possible the clinician in these scenarios felt the risk to benefit ratio favored continuing AVNBAs in these cases. Clinician decision‐making may have also factored into what kind of patients were selected for cardioversion after 2020 based on emerging evidence regarding the benefits of early rhythm control [11]. We entertained the possibility that the COVID‐19 pandemic influenced patient selection for elective cardioversion, but the trend for patient volumes (see Table S2) does not appear to bear this out as the years 2020–2022 show successively higher cardioversion volumes compared to previous years. We speculate this could be one factor why 30 day recurrent tachyarrhythmias were more frequent in the no‐hold group—arrhythmias that would previously have been resigned to rate control began receiving aggressive rhythm control. Additionally, although our regression model attempted to correct for baseline patient characteristics, potential unaccounted confounding and correlation among diagnoses may exist.

As a final note, despite this not being the main purpose of our study, our analysis is consistent with the generally accepted knowledge that, in appropriately selected patients, a DCCV is an effective and safe procedure. Our study demonstrated a 96% procedural success rate and only a single case where temporary pacing was needed. This also restricts our main analysis to group differences for potentially clinically insignificant bradyarrhythmia—mainly sinus bradycardia. New conduction disturbances outside of this were quite rare in the perioperative and 30 day follow up periods.

## 5 Conclusions

Our study demonstrates that holding AVNBAs for 24 h prior to DCCV was not significantly associated with perioperative conduction disorders and this remained the same after risk adjustment. Given the small number of mostly benign outcomes for patients included in this single center report, these findings should be verified by larger retrospective studies and a prospective randomized clinical trial.

## Funding

The authors have nothing to report.

## Ethics Statement

This research study was approved by the Providence Institutional Review Board.

## Consent

The authors have nothing to report.

## Conflicts of Interest

The authors declare no conflicts of interest.

## Supporting information


**Table S1:** Daily Total Metoprolol Dose by Time Points Relative to the Procedure.
**Table S2:** Number of Procedures in the AVNB Held and Not Held Groups by Year.
**Table S3:** Patient Characteristics Before and After the Practice Change.
**Table S4:** Patient Characteristics Among Procedures in 2021 and Beyond.

## Data Availability

The data that support the findings of this study are available from the corresponding author upon reasonable request.

## Appendix: ICD‐10 and CPT Codes A

**Table:**

Element	Codes
Heart Failure	*ICD‐10*: I09.81, I11.0, I13.0, I13.2, I50, I50.1, I50.2, I50.20, I50.21, I50.22, I50.23, I50.3, I50.30, I50.31, I50.32, I50.33, I50.4, I50.40, I50.41, I50.42, I50.43, I50.8, I50.81, I50.810, I50.811, I50.812, I50.813, I50.814, I50.82, I50.83, I50.84, I50.89, I50.9
Cardioversion	*ICD‐10*: 5A2204Z *CPT Code*: 92960, 92 961, 12 256 039, 12 256 039, 12 256 039, 12 256 039, 12 256 043, 12 256 043, 12 256 043, 12 256 043, 12 256 043, 122 235 573, 122 237 424
Conduction Disease	*ICD‐10*: I442, I440, I441, I4439, I455, I4430, I444, I447, I445, I452, I459, I4581, I454, I4469, I4589, I453, I4460, I4519, I450, I4510, I495
Diabetes	*ICD‐10*: E08, E08.0, E08.00, E08.01, E08.1, E08.10, E08.11, E08.2, E08.21, E08.22, E08.29, E08.3, E08.311, E08.319, E08.3211, E08.3212, E08.3213, E08.3219, E08.329, E08.3291, E08.3292, E08.3293, E08.3299, E08.331, E08.3311, E08.3312, E08.3313, E08.3319, E08.339, E08.3391, E08.3392, E08.3393, E08.3399, E08.341, E08.3411, E08.3412, E08.3413, E08.3419, E08.349, E08.3491, E08.3492, E08.3493, E08.3499, E08.351, E08.3511, E08.3512, E08.3513, E08.3519, E08.3521, E08.3522, E08.3523, E08.3529, E08.3531, E08.3532, E08.3533, E08.3539, E08.3541, E08.3542, E08.3543, E08.3549, E08.3551, E08.3552, E08.3553, E08.3559, E08.359, E08.3591, E08.3592, E08.3593, E08.3599, E08.36, E08.39, E08.4, E08.40, E08.41, E08.42, E08.43, E08.44, E08.49, E08.5, E08.51, E08.52, E08.59, E08.6, E08.610, E08.618, E08.620, E08.621, E08.622, E08.628, E08.630, E08.638, E08.641, E08.649, E08.65, E08.69, E08.8, E08.9, E09, E09.0, E09.00, E09.01, E09.1, E09.10, E09.11, E09.2, E09.21, E09.22, E09.29, E09.3, E09.311, E09.319, E09.321, E09.3211, E09.3212, E09.3213, E09.3319, E09.339, E09.3391, E09.3392, E09.3393, E09.3399, E09.341, E09.3411, E09.3412, E09.3413, E09.3419, E09.349, E09.3491, E09.3492, E09.3493, E09.3499, E09.351, E09.3511, E09.3512, E09.3513, E09.3519, E09.3521, E09.3522, E09.3523, E09.3529, E09.3531, E09.3532, E09.3533, E09.3539, E09.3541, E09.3542, E09.3543, E09.3549, E09.3551, E09.3552, E09.3553, E09.3559, E09.359, E09.3591, E09.3592, E09.3593, E09.3599, E09.36, E09.39, E09.4, E09.40, E09.41, E09.42, E09.43, E09.44, E09.49, E09.5, E09.51, E09.52, E09.59, E09.6, E09.610, E09.618, E09.620, E09.621, E09.622, E09.628, E09.630, E09.638, E09.641, E09.649, E09.65, E09.69, E09.8, E09.9, E10, E10.1, E10.10, E10.11, E10.2, E10.21, E10.22, E10.29, E10.3, E10.311, E10.319, E10.321, E10.3211, E10.3212, E10.3213, E10.3219, E10.329, E10.3291, E10.3292, E10.3293, E10.3299, E10.331, E10.3311, E10.3312, E10.3313, E10.3319, E10.339, E10.3391, E10.3392, E10.3393, E10.3399, E10.341, E10.3411, E10.3412, E10.3413, E10.3419, E10.349, E10.3491, E10.3492, E10.3493, E10.3499, E10.351, E10.3511, E10.3512, E10.3513, E10.3519, E10.3521, E10.3522, E10.3523, E10.3529, E10.3531, E10.3532, E10.3533, E10.3539, E10.3541, E10.3542, E10.3543, E10.3549, E10.3551, E10.3552, E10.3553, E10.3559, E10.359, E10.3591, E10.3592, E10.3593, E10.3599, E10.36, E10.39, E10.4, E10.40, E10.41, E10.42, E10.43, E10.44, E10.49, E10.5, E10.51, E10.52, E10.59, E10.6, E10.610, E10.618, E10.620, E10.621, E10.622, E10.628, E10.630, E10.638, E10.641, E10.649, E10.65, E10.69, E10.8, E10.9, E11, E11.0, E11.00, E11.01, E11.1, E11.10, E11.11, E11.2, E11.21, E11.22, E11.29, E11.3, E11.311, E11.319, E11.321, E11.3211, E11.3212, E11.3213, E11.3219, E11.329, E11.3291, E11.3292, E11.3293, E11.3299, E11.331, E11.3311, E11.3312, E11.3313, E11.3319, E11.339, E11.3391, E11.3392, E11.3393, E11.3399, E11.341, E11.3411, E11.3412, E11.3413, E11.3419, E11.349, E11.3491, E11.3492, E11.3493, E11.3499, E11.351, E11.3511, E11.3512, E11.3513, E11.3519, E11.3521, E11.3522, E11.3523, E11.3529, E11.3531, E11.3532, E11.3533, E11.3539, E11.3541, E11.3542, E11.3543, E11.3549, E11.3551, E11.3552, E11.3553, E11.3559, E11.359, E11.3591, E11.3592, E11.3593, E11.3599, E11.36, E11.39, E11.4, E11.40, E11.41, E11.42, E11.43, E11.44, E11.49, E11.5, E11.51, E11.52, E11.59, E11.6, E11.610, E11.618, E11.620, E11.621, E11.622, E11.628, E11.630, E11.638, E11.641, E11.649, E11.65, E11.69, E11.8, E11.9, E13, E13.0, E13.00, E13.01, E13.1, E13.10, E13.11, E13.2, E13.21, E13.22, E13.29, E13.3, E13.331, E13.3311, E13.3312, E13.3313, E13.3319, E13.339, E13.3391, E13.3392, E13.3393, E13.3399, E13.341, E13.3411, E13.3412, E13.3413, E13.3419, E13.349, E13.3491, E13.3492, E13.3493, E13.3499, E13.351, E13.3511, E13.3512, E13.3513, E13.3519, E13.3521, E13.3522, E13.3523, E13.3529, E13.3531, E13.3532, E13.3533, E13.3539, E13.3541, E13.3542, E13.3543, E13.3549, E13.3551, E13.3552, E13.3553, E13.3559, E13.359, E13.3591, E13.3592, E13.3593, E13.3599, E13.36, E13.39, E13.4, E13.40, E13.41, E13.42, E13.43, E13.44, E13.49, E13.5, E13.51, E13.52, E13.59, E13.6, E13.610, E13.618, E13.620, E13.621, E13.622, E13.628, E13.630, E13.638, E13.641, E13.649, E13.65, E13.69, E13.8, E13.9
Hypertension	*ICD‐10*: H35.031, H35.032, H35.033, I10, I11, I11.0, I11.9, I12, I12.0, I12.9, I13, I13.0, I13.1, I13.10, I13.11, I13.2, I15, I15.0, I15.1, I15.2, I15.8, I15.9, I16, I16.0, I16.1, I16.9, I67.4
Cerebrovascular Diseases	*ICD‐10*: G43.601, G43.609, G43.611, G43.619, G45.0, G45.1, G45.2, G45.3, G45.4, G45.8, G45.9, I63, I63.00, I63.01, I63.011, I63.012, I63.013, I63.019, I63.02, I63.03, I63.031, I63.032, I63.033, I63.039, I63.09, I63.10, I63.11, I63.111, I63.112, I63.113, I63.119, I63.12, I63.13, I63.131, I63.132, I63.133, I63.139, I63.19, I63.20, I63.21, I63.211, I63.212, I63.213, I63.219, I63.22, I63.23, I63.231, I63.232, I63.233, I63.239, I63.29, I63.30, I63.31, I63.311, I63.312, I63.313, I63.319, I63.32, I63.321, I63.322, I63.323, I63.329, I63.33, I63.331, I63.332, I63.333, I63.339, I63.34, I63.341, I63.342, I63.343, I63.349, I63.39, I63.40, I63.41, I63.411, I63.412, I63.413, I63.419, I63.42, I63.421, I63.422, I63.423, I63.429, I63.43, I63.431, I63.432, I63.433, I63.439, I63.44, I63.441, I63.442, I63.443, I63.449, I63.49, I63.50, I63.51, I63.511, I63.512, I63.513, I63.519, I63.52, I63.521, I63.522, I63.523, I63.529, I63.53, I63.531, I63.532, I63.533, I63.539, I63.54, I63.541, I63.542, I63.543, I63.549, I63.59, I63.6, I63.8, I63.81, I63.89, I63.9, I65, I65.0, I65.1, I65.2, I65.8, I65.9, I66, I66.0, I66.1, I66.2, I66.3, I66.8, I66.9, I69.30, I69.31, I69.32, I69.33, I69.34, I69.35, I69.36, I69.39, I97.81, I97.810, I97.811, I97.82, I97.820, I97.821, Z86.73, I63.0, I63.1, I63.2, I63.3, I63.4, I63.5, I69.310, I69.311, I69.312, I69.313, I69.314, I69.315, I69.318, I69.319, I69.320, I69.321, I69.322, I69.323, I69.328, I69.331, I69.332, I69.333, I69.334, I69.339, I69.341, I69.342, I69.343, I69.344, I69.349, I69.351, I69.352, I69.353, I69.354, I69.359, I69.361, I69.362, I69.363, I69.364, I69.365, I69.369, I69.390, I69.391, I69.392, I69.393, I69.398, I65.01, I65.02, I65.03, I65.09, I65.21, I65.22, I65.23, I65.29, I66.01, I66.02, I66.03, I66.09, I66.11, I66.12, I66.13, I66.19, I66.21, I66.22, I66.23, I66.29, I67.2
Atherosclerotic Cardiovascular Disease	*ICD‐10*: I71.3, I71.4, I24.0, I24.9, I21, I71.12, I71.22, I71.11, I71.21, I71.13, I71.23, Z98.6, I71, I71.8, I71.9, I70, I70.80, I25, I25.9, I70.92, I25.82, Z98.61, I25.41, I71.02, I71.0, I71.011, I71.010, I71.012, I71.01, I71.03, I71.00, I71.33, I71.43, I25.5, I71.32, I71.42, T82.21, I21.B, I25.2, I70.9, I70.49, I70.29, I70.59, I70.69, I70.79, I70.39, I25.89, I21.A9, I21.A, I71.31, I71.41, I71.52, I71.62, Z98.62, Z95.820, I23.7, Z95.1, Z95.9, Z95.9, Z95.5, Z95.818, Z95.828, I21.02, I21.21, I21.01, I21.09, I21.19, I21.29, I21.11, I22.2, I22.0, I22.1, I22.8, I22.9, I22, I71.51, I71.61, I71.1, I71.2, I71.5, I71.6, E10.52, E10.51, E11.52, E11.51, I71.019, I71.10, I71.20, I71.30, I71.40, I71.50, I71.60, I70.60, I20.0, I21.0, I21.1, I21.2, I21.3, I21.4, I21.9, I75.023, I75.013, I75.81, I75.022, I75.012, I75.02, I75.021, I75.011, I75.029, I75.019, I75.01, I25.751, I25.758, I25.759, I70.0, I25.72, I25.721, I25.728, I25.729, I25.720, I70.45, I70.4, I70.46, I70.463, I70.462, I70.468, I70.461, I70.469, I70.41, I70.413, I70.412, I70.418, I70.411, I70.419, I70.42, I70.423, I70.422, I70.428, I70.421, I70.429, I70.44, I70.443, I70.442, I70.444, I70.445, I70.448, I70.441, I70.449, I70.43, I70.433, I70.432, I70.434, I70.435, I70.438, I70.431, I70.439, I25.71, I25.711, I25.718, I25.719, I25.710, I25.768, I25.76, I25.761, I25.769, I25.760, I25.812, I25.7, I25.810, I25.70, I25.701, I25.708, I25.709, I25.700, I70.26, I70.263, I70.262, I70.268, I70.261, I70.269, I70.21, I70.213, I70.212, I70.218, I70.211, I70.219, I70.22, I70.223, I70.222, I70.228, I70.221, I70.229, I70.24, I70.243, I70.242, I70.244, I70.245, I70.248, I70.241, I70.249, I70.25, I70.23, I70.233, I70.232, I70.234, I70.235, I70.238, I70.231, I70.239, I70.2, I25.75, I25.750, I25.811, I70.55, I70.5, I70.51, I70.56, I70.563, I70.562, I70.568, I70.561, I70.569, I70.513, I70.512, I70.518, I70.511, I70.519, I70.52, I70.523, I70.522, I70.528, I70.521, I70.529, I70.54, I70.543, I70.542, I70.544, I70.545, I70.548, I70.541, I70.549, I70.53, I70.533, I70.532, I70.534, I70.535, I70.538, I70.531, I70.539, I25.73, I25.731, I25.738, I25.739, I25.730, I70.65, I70.6, I70.66, I70.663, I70.662, I70.668, I70.661, I70.669, I70.61, I70.613, I70.612, I70.618, I70.611, I70.619, I70.62, I70.623, I70.622, I70.628, I70.621, I70.629, I70.64, I70.643, I70.642, I70.644, I70.645, I70.648, I70.641, I70.649, I70.63, I70.633, I70.632, I70.634, I70.635, I70.638, I70.631, I70.639, I70.8, I25.798, I25.79, I25.791, I25.799, I25.790, I25.81, I70.75, I70.7, I70.76, I70.763, I70.762, I70.768, I70.761, I70.769, I70.71, I70.713, I70.712, I70.718, I70.711, I70.719, I70.72, I70.723, I70.722, I70.728, I70.721, I70.729, I70.74, I70.743, I70.742, I70.744, I70.745, I70.748, I70.741, I70.749, I70.73, I70.733, I70.732, I70.734, I70.735, I70.738, I70.731, I70.739, I70.1, I70.35, I70.3, I70.36, I70.363, I70.362, I70.368, I70.361, I70.369, I70.31, I70.313, I70.312, I70.318, I70.311, I70.319, I70.32, I70.323, I70.322, I70.328, I70.321, I70.329, I70.34, I70.343, I70.342, I70.344, I70.345, I70.348, I70.341, I70.349, I70.33, I70.333, I70.332, I70.334, I70.335, I70.338, I70.331, I70.339, I25.0, I25.11, I25.111, I25.118, I25.119, I25.110, I25.1, I25.10, I67.2, I25.84, I25.83, I70.91, I70.49, I70.493, I70.492, I70.498, I70.491, I70.499, I70.29, I70.293, I70.292, I70.298, I70.291, I70.299, I70.59, I70.593, I70.592, I70.598, I70.591, I70.599, I70.69, I70.693, I70.692, I70.698, I70.691, I70.699, I70.79, I70.793, I70.792, I70.798, I70.791, I70.799, I70.39, I70.393, I70.392, I70.398, I70.391, I70.399, I70.90, I70.40, I70.403, I70.402, I70.408, I70.401, I70.409, I70.20, I70.203, I70.202, I70.208, I70.201, I70.209, I70.50, I70.503, I70.502, I70.508, I70.501, I70.509, I70.60, I70.603, I70.602, I70.608, I70.601, I70.609, I70.70, I70.703, I70.702, I70.708, I70.701, I70.709, I70.30, I70.303, I70.302, I70.308, I70.301, I70.309
Pacemaker/Defibrillator	*ICD‐10*: 02H40JZ, 02H40KZ, 02H40MZ, 02H40NZ, 02H43JZ, 02H43KZ, 02H43MZ, 02H43NZ, 02H44JZ, 02H44KZ, 02H44MZ, 02H44NZ, 02H60JZ, 02H60KZ, 02H60MZ, 02H60NZ, 02H63JZ, 02H63KZ, 02H63MZ, 02H63NZ, 02H64JZ, 02H64KZ, 02H64MZ, 02H64NZ, 02H70JZ, 02H70KZ, 02H70MZ, 02H70NZ, 02H73JZ, 02H73KZ, 02H73MZ, 02H73NZ, 02H74JZ, 02H74KZ, 02H74MZ, 02H74NZ, 02HK0JZ, 02HK0KZ, 02HK0MZ, 02HK0NZ, 02HK3JZ, 02HK3KZ, 02HK3MZ, 02HK3NZ, 02HK4JZ, 02HK4KZ, 02HK4MZ, 02HK4NZ, 02HL0JZ, 02HL0KZ, 02HL0MZ, 02HL0NZ, 02HL3JZ, 02HL3KZ, 02HL3MZ, 02HL3NZ, 02HL4JZ, 02HL4KZ, 02HL4MZ, 02HL4NZ, 02HN0JZ, 02HN0KZ, 02HN0MZ, 02HN3JZ, 02HN3KZ, 02HN3MZ, 02HN4JZ, 02HN4KZ, 02HN4MZ, 0JH604Z, 0JH605Z, 0JH606Z, 0JH607Z, 0JH609Z, 0JH634Z, 0JH635Z, 0JH636Z, 0JH637Z, 0JH639Z, 0JH63PZ, 0JH804Z, 0JH805Z, 0JH806Z, 0JH807Z, 0JH808Z, 0JH809Z, 0JH80PZ, 0JH835Z, 0JH836Z, 0JH837Z, 0JH838Z, 0JH839Z, 0JH83PZ, Z45010, Z45018, Z950. *CPT Code*: 0387 T, 0388 T, 0416 T, 0515 T, 0516 T, 0517 T, 0518 T, 0519 T, 0520 T, 0571 T, 0572 T, 0573 T, 0574 T, 0580 T, 0614 T, 33202, 33 203, 33 206, 33 207, 33 208, 33 210, 33 211, 33 212, 33 213, 33 214, 33 215, 33 216, 33 217, 33 218, 33 220, 33 221, 33 222, 33 223, 33 224, 33 226, 33 227, 33 228, 33 229, 33 230, 33 231, 33 233, 33 234, 33 235, 33 236, 33 237, 33 238, 33 240, 33 241, 33 243, 33 244, 33 249, 33 262, 33 263, 33 264, 33 274, 33 275, G0448
Sleep Apnea	*ICD‐10*; G4730, G4731, G4733, G4737, G4739, G473, G4734, G4735, G4736
