# Supplementary material for: Atrioventricular Nodal Blocking Agents and Adverse Outcomes Following Cardioversion at a Rural Hospital
Source: J Arrhythm. 2026 Oct 23;42(5):e70481. doi: 10.1002/joa3.70481 (PMC13691538; doi:10.1002/joa3.70481)
Supplement: Supplementary file 1 [file JOA3-42-e70481-s001.docx]

***Journal of Arrhythmia***

**Atrioventricular Nodal Blocking Agents and Adverse Outcomes Following Cardioversion at a Rural Hospital**

Zach Caverley, MSBS, PA-C^a^, Hsin-Fang Li, PhD^b^, Kateri J Spinelli, PhD^b^

a. Providence Heart Clinic North Coast, Providence Oregon Cardiology Group, Seaside, Oregon

b. Center for Cardiovascular Analytics, Research and Data Science (CARDS), Providence Heart Institute, Providence Research Network, Portland, Oregon

Corresponding Author: Zach Caverley, MSBS, PA-C
Email: Zachary.caverley@providence.org

Address: 725 S Wahanna Rd, Seaside, OR 97138

Business phone: 503-717-7850 / Fax: 503-216-0920

**Supplemental Appendix**

**Supplementary Table 1.** Daily Total Metoprolol Dose by Time Points Relative to the Procedure

**Supplementary Table 2.** Number of Procedures in the AVNB Held and Not Held Groups by Year

**Supplementary Table 3.** Patient Characteristics Before and After the Practice Change

**Supplementary Table 4.** Patient Characteristics Among Procedures in 2021 and Beyond

Supplementary Table 1. Daily Total Metoprolol Dose by Time Points Relative to the Procedure

| **Medication** | **2-week pre-op** | **72hrs pre-op** | **Discharge** | **2-week post-op** | **30-day post-op** |
| --- | --- | --- | --- | --- | --- |
| **All Procedure** |  |  |  |  |  |
| Metoprolol tartrate | 100 (50, 150) | 100 (50, 150) | 75 (50, 100) | 0 (0, 0) | 100 (50, 100) |
| Metoprolol succinate | 75 (50, 100) | 87.5 (50, 100) | 50 (25, 100) | 50 (25, 100) | 50 (25, 100) |
| **AVNBA Not Held** |  |  |  |  |  |
| Metoprolol tartrate | 100 (50, 150) | 100 (50, 200) | 50 (50, 150) | 100 (50, 150) | 100 (50, 150) |
| Metoprolol succinate | 87.5 (50, 200) | 100 (25, 200) | 100 (50, 150) | 62.5 (25, 200) | 50 (25, 100) |
| **AVNBA Held** |  |  |  |  |  |
| Metoprolol tartrate | 100 (75, 150) | 100 (100, 150) | 100 (50, 100) | 75 (50, 100) | 100 (50, 100) |
| Metoprolol succinate | 50 (50, 100) | 62.5 (50, 100) | 50 (25, 100) | 50 (25, 100) | 50 (25, 100) |

Data presented as median [interquartile range].

Abbreviations: AVNBA = atrioventricular nodal blocking agents

**Supplementary Table 2**. Number of Procedures in the AVNBA Held and Not Held Groups by Year

|  | **AVNBA Not Held** (N=150) | **AVNBA Held** (N=97) | **AVNBA Not Held** (N=150) | **AVNBA Held** (N=97) |
| --- | --- | --- | --- | --- |
| **Year** | Column Percentage (%) | | Row Percentage (%) | |
| 2016 | 10 (7%) |  | 10 (100%) |  |
| 2017 | 11 (7%) |  | 11 (100%) |  |
| 2018 | 20 (13%) |  | 20 (100%) |  |
| 2019 | 18 (12%) | 3 (3%) | 18 (86%) | 3 (14%) |
| 2020 | 37 (25%) |  | 37 (100%) |  |
| 2021 | 39 (26%) | 14 (14%) | 39 (74%) | 14 (26%) |
| 2022 | 9 (6%) | 47 (48%) | 9 (16%) | 47 (84%) |
| 2023 | 6 (4%) | 33 (34%) | 6 (15%) | 33 (85%) |

Data presented as n (%).

Abbreviations: AVNBA = atrioventricular nodal blocking agents

**Supplementary Table 3.** Patient Characteristics for the AVNBA Not Held Group Before and After 2020.

| **Variable** | **N missing** | **AVNBA Not Held** | | |
| --- | --- | --- | --- | --- |
|  |  | **≤2020** | **2021+** | **P-value comparing  ≤2020 vs. 2021+** |
| Number of patients | N/A | 96 | 54 | N/A |
| Age, years | 0 | 68.4 (9.9) | 72.5 (8.5) | 0.045 |
| Sex, female | 0 | 67 (70%) | 34 (63%) | 0.4 |
| BMI | 0 | 34.5 (8.3) | 32.2 (6.3) | 0.079 |
| Race, White | 15 | 84 (88%) | 48 (89%) | 0.8 |
| Insurance Type | 0 |  |  | 0.1 |
| Commercial |  | 16 (17%) | 3 (5.6%) |  |
| Medicaid/low-income |  | 2 (2.1%) | 3 (5.6%) |  |
| Medicare |  | 71 (74%) | 47 (87%) |  |
| Self-pay |  | 2 (2.1%) | 0 (0%) |  |
| Other |  | 5 (5.2%) | 1 (1.9%) |  |
| Baseline Comorbidities |  |  |  |  |
| Heart failure | 0 | 28 (29%) | 11 (20%) | 0.2 |
| Hypertension | 0 | 72 (75%) | 41 (76%) | 0.9 |
| Diabetes mellitus | 0 | 17 (18%) | 9 (17%) | 0.9 |
| Sleep apnea | 0 | 34 (35%) | 15 (28%) | 0.3 |
| Prior or multiple  Cardioversion | 0 | 6 (6.3%) | 6 (11%) | 0.4 |
| Pacemaker/ICD | 0 | 5 (5.2%) | 3 (5.6%) | 1 |
| ASCVD |  | 29 (30%) | 14 (26%) | 0.6 |

Data presented as n (%).

Abbreviations: ASCVD = atherosclerotic cardiovascular disease, AVNBA = atrioventricular nodal blocking agents, BMI = body mass index, ICD = implantable cardioverter-defibrillator

**Supplementary Table 4**. Patient Characteristics For Procedures in 2021 and Beyond.

| **Variable** | **N missing** | **AVNBA Not Held** | **AVNBA Held** | **p-value** |
| --- | --- | --- | --- | --- |
| Number of patients | N/A | 54 | 94 | N/A |
| Age, years | 0 | 72.5 (8.5) | 71.8 (8.1) | 0.8 |
| Sex, female | 0 | 34 (63%) | 66 (70%) | 0.4 |
| BMI | 0 | 32.2 (6.3) | 32.7 (7.1) | 0.7 |
| Race, White | 15 | 48 (89%) | 89 (95%) | 0.2 |
| Insurance Type | 0 |  |  | 0.054 |
| Commercial |  | 3 (5.6%) | 17 (18%) |  |
| Medicaid/low-income |  | 3 (5.6%) | 1 (1.1%) |  |
| Medicare |  | 47 (87%) | 73 (78%) |  |
| Other |  | 1 (1.9%) | 3 (3.2%) |  |
| Baseline Comorbidities |  |  |  |  |
| Heart failure | 0 | 11 (20%) | 18 (19%) | 0.9 |
| Hypertension | 0 | 41 (76%) | 68 (72%) | 0.6 |
| Diabetes mellitus | 0 | 9 (17%) | 12 (13%) | 0.5 |
| Sleep apnea | 0 | 15 (28%) | 29 (31%) | 0.7 |
| Prior or multiple  cardioversion | 0 | 6 (11%) | 6 (6.4%) | 0.4 |
| Pacemaker/ICD | 0 | 3 (5.6%) | 7 (7.4%) | 0.7 |
| ASCVD |  | 14 (26%) | 35 (37%) | 0.2 |

Data presented as n (%).

Abbreviations: ASCVD = atherosclerotic cardiovascular disease, AVNBA = atrioventricular nodal blocking agents, BMI = body mass index, ICD = implantable cardioverter-defibrillator
